# Research on the Intrinsic Sensing Performance of an Optical Fiber Dosimeter Based on Radiation-Induced Attenuation

**DOI:** 10.3390/s25123716

**Published:** 2025-06-13

**Authors:** Junyu Hou, Zhanzu Feng, Ge Ma, Weiwei Zhang, Zong Meng, Yuhe Li

**Affiliations:** 1 Department of Precision Instruments, Tsinghua University, Beijing 100084, China; houjy19@mails.tsinghua.edu.cn; 2 School of Nuclear Science and Technology, Lanzhou University, Lanzhou 730000, China; fengzhz2023@lzu.edu.cn; 3 Department of Engineering Physics, Tsinghua University, Beijing 100084, China; mg20@mails.tsinghua.edu.cn; 4 School of Electrical Engineering, Yanshan University, Qinhuangdao 066004, China; zhangww@stumail.ysu.edu.cn (W.Z.); mzysu@ysu.edu.cn (Z.M.)

**Keywords:** radiation-induced attenuation, optical fiber dosimeter, sensing performance

## Abstract

Current research on dosimeters based on radiation-induced attenuation (RIA) primarily focused on enhancing radiation sensitivity or reducing dependencies from interference factors. However, their intrinsic sensing performance has received limited attention. This work proposed application and analysis methods for RIA-based dosimeters, validated by a low-cost apparatus using commercial fibers. Initially, a generic protocol of high-dose detection after low-dose calibration was suggested to overcome the various dependencies of RIA, enabling repetitive monitoring of near-stable radiation by simple replacement of commercial fibers. Experiments comparing three dose-loss models demonstrated that the saturation-exponential model exhibited superior accuracy, achieving absolute errors below 4 Gy within a measurable range of up to ~300 Gy. Subsequently, the system’s RIA-based sensitivity was ~125.6 dB·Gy^−1^·km^−1^. The resolution and sensitivity expressed by optical power were newly defined, effectively quantifying the decline in precision and response ratio during detection. Moreover, an additional structure was introduced to extend the measurable range. Simulations and experiments under 1-MeV electron irradiation verified that adjustable ranges could be achieved through configuration of attenuation layers. In summary, these advancements provided critical guidance for component selection and operational evaluation, facilitating the commercialization and practical deployment of RIA-based dosimeters.

## 1. Introduction

Optical fibers have been increasingly deployed in nuclear facilities and space systems [1,2] to monitor temperature or other parameters, owing to their advantages such as electromagnetic interference immunity, compact size, lightweight design, and distributed sensing capabilities. Researchers have concurrently focused on radiation-induced alterations in fiber performance [3]. Specifically, radiation degrades the fiber’s transmission properties, and radiation-induced attenuation (RIA), which could be measured optically, exhibited a predictable relationship with cumulative radiation dose [4].

The causation of RIA in silica fibers has been investigated since the 1960s [5,6,7], forming foundational insights into radiation effects on fiber properties. A number of review papers [8,9,10,11,12] systematically summarized the mechanisms of structural modifications in diverse dopant fibers under radiation, while defect centers were widely employed to illustrate RIA. Point defects, including E’ centers, oxygen deficiency centers (ODCs), and peroxyl radicals (PORs), were inherently formed in silica fibers. To meet the requirement of low-loss propagation, dopants (e.g., Ge, F) and impurities (e.g., Cl^−^, OH^−^) also persisted due to the fabrication methods or environments [1]. Although advanced manufacturing has reduced impurity concentrations to parts-per-billion (ppb) levels, intrinsic defects and dopants in fibers remained unavoidable [13]. High-energy radiation caused ionization damage and displacement damage to the fibers, which generated electron-hole pairs, vacancies, and interstitial atoms [3]. Atomic vacancies and interstitial atoms induced rearrangement of the atomic structure, which could also trigger displacement of inherent structure and accelerate generation of point defects. Charged carriers (e.g., electrons, holes) were trapped by precursor defects, forming defects with stabilized structures [14]. These processes enhanced pre-existing localized states or introduced new ones within the bandgap. And the additional light absorption at different wavelengths for electronic transitions constituted the characteristic spectrum of RIA. A type of point defect might correlate with absorption peaks at multiple wavelengths, and studies mentioned before [1,5,6,7,8,9,10,11,12,13,14] have also been devoted to their optical properties, thermal stability, and photostability. Current research on fiber radiation characteristics remained concentrated on elucidating point defect properties [1,15] conducting multi-scale simulations [16,17], developing and testing advanced fibers (e.g., radiation-hardened fibers [18,19] and radiation-sensitive fibers [20,21]), and investigating radiation-induced interference in diverse fiber sensors [22,23].

The concept of leveraging RIA for radiation detection was repeatedly proposed throughout the 1980s [24]. By 1994, Los Alamos National Laboratory (LANL) embedded Ge-doped multimode fibers within the surface structures of a satellite, establishing an RIA relationship with doses of X-ray radiation up to ~10 Gy [25]. Over the past two decades, research in this field has remained predominantly focused on advancing the composition of the core or cladding [15] for novel radiation-sensitive fibers, aimed at optimizing their radiation-induced characteristics. However, this task is non-trivial, because a perfect sensing fiber for RIA-based dosimeters must exhibit strictly monotonic dose dependence of RIA, negligible spontaneous recovery post-irradiation, dose rate independence, and the immunity to thermal- or photobleaching [26]. Pure silica cores and F-doped cladding fibers alway showed lower radiation sensitivity for stable irradiation, but had the strongest RIA level after pulsed X-ray irradiation [27]. Comparable to the pure silica core fiber response, N-doped fibers presented good radiation resistance at low doses for both transient and stable irradiation [15]. Al-doped fibers could retain an identical RIA within about 15% while the dose rate changed in 0.073 to 6.25 Gy/s, but a higher dependence on temperature was observed [28]. Furthermore, no specific absorption dependent on the Al content could be discriminated in the NIR [29]. Ge-doped fibers have been deeply evaluated, since most of telecom-grade waveguides were of this type [1]. Ge-related defects were more efficiently generated than Si-related defects [30], but most Ge-related fibers exhibited varying degrees of temperature [31] and dose rate [32] dependency. P-doped fibers have been already proven to be good candidates for fiber dosimetry; high P-doped step-index multimode fibers revealed low recovery and reduced dose rate dependency under γ-ray irradiation at dose rates exceeding 1 Gy/h [33,34]. In recent years, S. Girard, D.D. Francesca, G.L. Vecchi, and their collaborators, the leading research group in the development of P-doped fibers, have engineered single-mode fibers that exhibited negligible sensitivity to dose rate, temperature, and irradiation history, along with a linear RIA–dose relationship up to 500 Gy [21,35]. These fibers enabled RIA recovery of about ~97% through photobleaching (PB) while maintaining a consistent response during reuse [36]. Leveraging optical time-domain reflectometry (OTDR), dosimeters based on these fibers have been deployed for distributed radiation monitoring at the European Organization for Nuclear Research (CERN) and the International Space Station (ISS) [2,37]. Beyond single-dopant fibers, Ge/P co-doped fibers, whether in single-mode or multimode configurations, emerged as promising candidates for optical fiber dosimeters [26,38]. In addition to silica optical fibers, polymethylmethacrylate (PMMA)-based plastic optical fibers (POFs) have also demonstrated predictable patterns of RIA [39,40], and co-extruded perfluorinated polymer optical fibers (PF-POFs) have been employed in RIA-based dosimeters, despite exhibiting distinct temperature and dose-rate dependencies [4].

The development of novel doped fibers with superior reliability and sensitivity was pivotal for advancing the sensing performance of RIA-based dosimeters. However, novel doped fibers for radiation detection remain in the preparation and verification stages in the laboratory currently, making them difficult to obtain commercially. Most fibers, especially commercial telecom-grade ones, exhibited various dependencies under radiation, limiting their application in RIA-based fiber dosimeters. Additionally, existing analytical methods predominantly focused on characterizing the radiation response properties of novel radiation-sensitive fibers while paying little attention to the inherent characteristics of the sensors. Therefore, we proposed an implementation method of low-dose calibration followed by high-dose detection under identical irradiation conditions, enabling RIA-based dosimeters constructed by even commercial fibers to achieve reliable measurements in near-stable irradiation. Using commercial fibers and optical power meters, we built a verification system. Its detection accuracy, the sensitivity traceable to optical powers, the resolution, and the repeatability of the same fiber type were analyzed from the perspective of sensors. These metrics visually demonstrated response variations during operation, providing new references for performance evaluation. We further actively introduced attenuation layers to extend the detectable ranges without changing the sensing fiber type. Experiments showed promising detection performance and scalability, proving its potential as an interesting candidate for repetitive measurements in predefined scenarios like food irradiation, radiotherapy, and accelerator monitoring. Though this disposable approach limited the complexity of application scenarios, it fully leveraged the affordability, maturity, user-friendliness, and commercial availability of common fibers, significantly reducing the difficulty and cost of building RIA-based dosimeters while facilitating their commercialization.

## 2. Application and Analytical Methods

### 2.1. Application Methods

The RIA could be calculated by Equation (1) during irradiation:

(1) $$RIA\left(t,\lambda \right)=-\frac{10}{l_{0}}lg\frac{P\left(t,\lambda \right)}{P\left(0,\lambda \right)}$$

where *P* is the power of the transmitted light measured at any time *t* and any wavelength λ, with *t* = 0 as the irradiation start. *l*_0_ was the exposure length of the sensing fibers.

The RIA levels and kinetics of a given silica fiber depended on numerous factors: (1) the radiation parameters, such as dose [41,42], dose rate [32,43], temperature [35,44], and injected light power [45,46]; and (2) the intrinsic characteristics of fibers, such as the type and concentration of dopants [27,47] and the manufacturing process [48]. Accurately predicting RIA levels under variable parameters remained challenging due to multiple dependencies. However, when radiation parameters and experimental conditions were quasi-stable, and a specific fiber type was predetermined, the dose–RIA relationship demonstrated quantifiable predictability. Under such a setting, empirical or semi-empirical models have been developed through mathematical fitting and kinetic formulations [49] of the first [50], second [51], or higher [52] order. These power [53], stretched exponential [54], saturating exponential [55], or more complex [56,57] models predicted RIA or loss as a function of cumulative doses, effectively matching the degradation of transmitted optical powers under radiation.

The predictability of RIA offered a feasible application method: after defining the target radiation environment and requirements (e.g., approximate dose rate and total dose), a type of doped fibers could be selected as the sensing fibers in the dosimeter, ensuring that the maximum RIA-correlated dose range meet the needs of detection. Initially, the optical power *P*(0) at a selected wavelength transmitted in each fiber was measured and recorded before all irradiation phases. Prior to deployment, these fibers were exposed to low-dose irradiation (e.g., ≤10% of the target dose) in the target radiation environment. Concurrently, real-time dose data of radiation sources were logged alongside the measured values of RIA, enabling model fitting between them. These stages concurrently served as calibration for each sensing fiber. During deployment, users could convert real-time optical power to RIA via Equation (1). These values of RIA were then input into the pre-calibrated model to derive real-time estimates of cumulative dose. However, these estimates included the doses during calibration. Thus, subtracting the estimate at the start of detection from these estimates, the real-time radiation doses outputted by RIA-based dosimeters could be calculated. This calibration-application workflow shared similar principles with radiation-sensing field-effect transistors (RADFETs) [58,59].

This application method might enable commercial fibers to serve as viable candidates for RIA-based dosimeters. However, it restricted application scenarios to near-stable radiation environments, such as long-term monitoring of accelerators or nuclear facilities during steady-state operation, or dose detection during batch irradiation sterilization of food using stable sources. After each measurement, the system could be reset by simply replacing the fiber—a process with minimal operational complexity and low cost.

### 2.2. Detection Accuracy

As discussed in the preceding method, the accuracy of dose detection critically depended on whether the fitted model after calibration could reliably predict RIA within extended dose ranges. This is particularly relevant, given that RIA often exhibited saturation trends at high-dose ranges, which could lead to rapidly increasing prediction errors. Additionally, for practical engineering applications, the model must balance accuracy with computational simplicity to avoid prohibitive processing requirements. Therefore, during feasibility validation, it is advisable to prioritize established models that have demonstrated acceptable fitting accuracy while maintaining relative simplicity to predict RIA. Thanks to prior deductions and validations [49,50,51,52,53,54,55,56,57], we adopted the power law model [53], as shown in Equation (2), which has demonstrated superior predictive accuracy for Ge-doped fibers [1]:

(2) $$RIA=c\times D^{f}+g$$

where *D* was the radiation dose, and *c* and *f* were the experimental constants. *g* was introduced as a compensation constant for zero-bias correction. Due to the saturation of RIA, the parameter typically satisfied 0 < *f* < 1.

Furthermore, a saturation-exponential model [49] was employed, as expressed in Equation (3), established through first-order kinetic formulations for defect generation and recovery during near-stable radiation:

(3) $$RIA=\varepsilon (1-e^{\frac{h-D}{d}})$$

where *d* is a constant related to saturation dose, *ε* is the proportional amplification coefficient, and *h* is also a compensation constant for zero-bias correction. Naturally, the simplest linear model was also incorporated into consideration.

Beyond model selection, the validation experiments still required meticulous design. Specifically, the radiation environments during the calibration and detection phases must maintain consistency, and a radiation-free interval should be incorporated between two phases to evaluate potential interfering factors such as RIA recovery and their impact on detection phases.

### 2.3. Sensitivity and Resolution

Sensitivity was defined as the change in output caused by unit variation of the measured quantity. Previous studies [4,21] predominantly focused on evaluating the sensibility or tolerance of novel fibers under radiation. Since RIA calculated via Equation (1) eliminated confounding factors like fiber length, it provided a more accurate and comparable metric for these characteristics. Consequently, researchers commonly utilized RIA-based sensitivity, defined as Equation (4):

(4) $$S_{RIA}=\frac{dRIA}{dD}$$

However, S*_RIA_* always remained approximately constant, or exhibited at most marginal decay within dose ranges far less than the saturation dose of the sensitive fiber. And this near-linear behavior underpinned the widespread use of the slope from the linear model to characterize S*_RIA_*, either to highlight enhanced radiation responsivity in advanced fibers or to validate dose-response uniformity within extended dose ranges.

It was critical to note that the raw output of an RIA-based dosimeter was the optical power *P*, as RIA or transmission loss was derived from real-time and pre-irradiation data via Equation (1). Therefore, sensitivity could alternatively be defined using optical power, expressed mathematically in Equation (5):

(5) $$S_{P}=\frac{dP}{dD}$$

Unlike RIA-based sensitivity, power-based sensitivity, which is untreated by Equation (1), avoided nonlinear conversion in RIA derivation, thereby uncovering distinct response phenomena.

Resolution, defined as the minimum detectable variation that a sensor could reliably discern and output, was fundamentally constrained by noise fluctuations and quantization errors. Similarly, these factors directly perturbed the measurements of raw optical power. Critically, such real-time perturbations were amplified through fitting models, ultimately limiting the minimum resolvable interval of doses. Thus, resolution could be quantified via Equation (6):

(6) $$∆D=\frac{dD}{dP}\times ∆P$$

where Δ*P* was the minimum resolvable interval of the optical power, constrained by noise and quantization errors. The resolution calculated by Equation (6) was expected to reveal the detection precision of the dosimeter at different times or doses.

### 2.4. Detectable Range

For commercial fibers without radiation-hardening treatments, the maximum dose which demonstrated predictable RIA behavior typically resided around 10^3^ Gy [1,41,42]. Beyond this threshold, RIA exhibited unpredictable variations, or even anomalous decreases. However, applications such as accelerator beam monitoring and material irradiation testing often required dose measurements far exceeding 10^3^ Gy. Continued use of these commercial fibers under such high-dose conditions risked failure of fitting models, severely compromising the detection performance of dosimeters. At elevated radiation doses, maintaining predictable RIA variations necessitated limiting the total energy deposition in the sensing fiber to low levels. This could be achieved by reducing the proportion of incident particles reaching the sensing fibers. In this work, we proposed emplacing configurable attenuation layers to enable adjustable detection ranges while retaining the original fiber type.

Under irradiation of identical and evenly distributed beam, the energy deposition in a bare sensing fiber was denoted as *E_a_*, and *E_p_* represented that in a sensing fiber of equal length and the same type encapsulated within an attenuation layer. The transmission *K_t_* of the attenuation layer could be defined using Equation (7):

(7) $$K_{t}=\frac{E_{P}}{E_{a}}$$

The original detection range of the dosimeter without attenuation layers under a kind of radiation environment was denoted as *R_o_*, indicating that the RIA of the sensing fiber reached its usable upper limit at this dose. Assuming a similar detectable threshold for RIA existed in fibers of the same type, the detection range *R_e_* with the attenuation layer under the same radiation environment could be estimated using Equation (8):

(8) $$R_{e}\approx \frac{R_{o}}{K_{t}}$$

Although the energy deposition in the sensing fiber was reduced, pairing between environmental doses and the RIA still remained. Model fitting and decoupling under proportional attenuation enabled adjustable detection ranges in our dosimeters. The approximation symbol ≈ in Equation (8) was intentional due to two factors: (1) even among fibers of the same type, slight variations in the detectable threshold of RIA introduced inevitable deviation in *R_e_*; and (2) most commercial fibers exhibited dose rate dependency, where RIA accumulation decreased at lower dose rates [32,33,34,42]. Since attenuation layers might reduce the dose rate to some extent, the actual RIA growth rate might also become slower than the product of *K_t_* and the unattenuated rate. Consequently, the practical detection range with such an attenuation layer might exceed *R_e_* as estimated using Equation (8).

By selecting attenuation layers with distinct geometries, materials, and thicknesses, the dosimeter could be equipped with varying *K_t_* values, thereby enabling multiple options of detection ranges. Thus, for applications in diverse radiation environments, targeted simulation or experimental validation of attenuation layer configurations became essential. This approach avoided fiber-type changes or fiber retesting, offering a more efficient and reliable pathway for range adaptation.

## 3. Experimental Details

### 3.1. Construction of the Validation Apparatus

**Figure 1:**
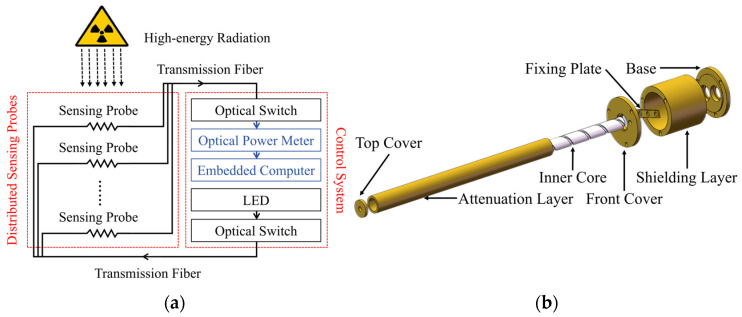
Schematic illustration of the validation apparatus used for online dose measurement. (**a**) Composition of the validation apparatus. (**b**) Structure of the sensing probe.

To validate the mentioned application and analytical methods, a low-cost, compact detection apparatus was constructed, serving as a prototype of the RIA-based dosimeter. As shown in Figure 1a, this detection apparatus comprised the sensing probes and the control system.

The sensing probes primarily functioned to secure a 0.5 m long sensing fiber, with its structure detailed in Figure 1b. The cylindrical structure of the inner core enabled omnidirectional radiation reception. The attenuation layer regulated radiation transmission to adjust the detection range. The shielding layer with enhanced thickness minimized interference at fiber connectors under radiation. The base integrated two fiber couplers to connect the sensing fibers in the probe and external long-distance transmission fibers. The front-end core region of the sensing fibers measured 150 mm (L) × 6 mm (W) × 6 mm (H). Aluminum was selected for the material of internal components to reduce weight, while copper for the outer casing was selected to maintain shielding effectiveness.

The sensing fiber was selected as OM2 graded-index (GRIN) fibers produced by Corning Incorporated (Corning, NY, USA), an industry-standard telecom-grade multimode fiber with a Ge dopant. In Table 1, we reported the useful specifics given by the manufacturer. The transmission fiber retained the same fiber but was clad with a thickened armored layer and supplemented with shielding blocks to mitigate the impact of scattered high-energy particles. To minimize potential high transmission losses in the ultraviolet band by these fibers, the verification apparatus adopted a 625 nm light source. Relevant studies have confirmed significant RIA at this wavelength, primarily caused by defect centers such as non-bridging oxygen hole centers (NBOHCs), peroxy radicals (PORs), and Ge-NBOHC [15,42,60]. These defect centers exhibited dose rate dependency and recovery under high temperatures to varying degrees [42], which provided an opportunity to validate the feasibility of the aforementioned application method.

Although LEDs exhibited an extended lifespan, their optical power might degrade due to thermal accumulation. To mitigate this, Fiber-coupled LED M625F2 (including LED Driver LEDD1B) from Thorlabs (Newton, NJ, USA) was affixed to a honeycomb-structured exoergic material and thermally coupled to the casing of the control system for enhanced heat dissipation. To further stabilize the optical power, a warm-up period was allocated to ensure that the 10 s averaged output remained virtually unchanged over 60 min prior to the experiments. The optical power meter PM100USB and slim photodiode sensor S130C (including thread adapter SM1A29 SM1) were also from Thorlabs (Newton, NJ, USA), achieving a lower detection limit of 500 pW and a resolution of 100 pW at 625 nm. The optical switches were optional components, enabling the control system to interface with multiple sensing probes and thereby facilitate the capability of simplified distributed detection.

### 3.2. Description of Experiments

The irradiation experiments were performed using the high-precision electron accelerator at the Lanzhou Institute of Physics. The energy of electron radiation could be adjusted within 0–2 MeV, and the dose rate could be adjusted from roughly 10^2^ Gy(SiO_2_)/s down to 10^−5^ Gy/s. With professional calibration, it exhibited an energy deviation ≤2% and a current instability ≤3%. Equipped with a Faraday cup and current integrator, its output data with a measurement error of ≤2% could be used for comparison and calibration. The facility could deliver a collimated particle beam with a diameter of ~300 mm. The irradiation room was positioned on the third basement level, with the temperature stable between 15 and 17 °C throughout the experiment.

In this experiment, to mitigate recovery from photobleaching, the LED provided constant optical power to the sensing fibers within a range of 10^−5^ W to 10^−6^ W [46]. In the experimental analysis, the primary data utilized were the optical power *P* measured at any time *t* and the loss of transmission light *L*, calculated using Equation (9):

(9) $$L\left(t\right)=-10lg\frac{P\left(t\right)}{P\left(0\right)}$$

The RIA calculated from Equation (1) and *L* derived from the same data only exhibited a linear relationship expressed by *L* = RIA·*l*_0_. After consolidation of this fixed scaling coefficient, the *D*-*L* models could have the same structural configuration of expression with the *D*-RIA models, as shown in Section 2.2. Therefore, substituting *L* for RIA preserved the validity of both the preliminary investigations and the subsequent analytical conclusions.

If optical switches were employed to cyclically measure the powers of multiple sensing fibers, the measurement light effectively acted as a low-frequency pulsed source for each fiber. Under such conditions, the photobleaching dynamics would change [46], necessitating additional experiments for analysis.

## 4. Analysis and Results

### 4.1. Detection Accuracy

The experiment was conducted without an attenuation layer, with the irradiated electron energy set to 1 MeV and the dose rate stabilized at ~0.498 Gy/s. The first irradiation phase lasted 54 s, delivering a cumulative dose of 27 Gy. This phase served as calibration for the dosimeter. Following irradiation, the dosimeter remained idle in a radiation-free environment for ~7 h. Subsequently, a second irradiation phase under identical radiation conditions was performed for 613 s, achieving a cumulative dose of 305 Gy, which corresponded to the detection phase of the dosimeter.

**Figure 2:**
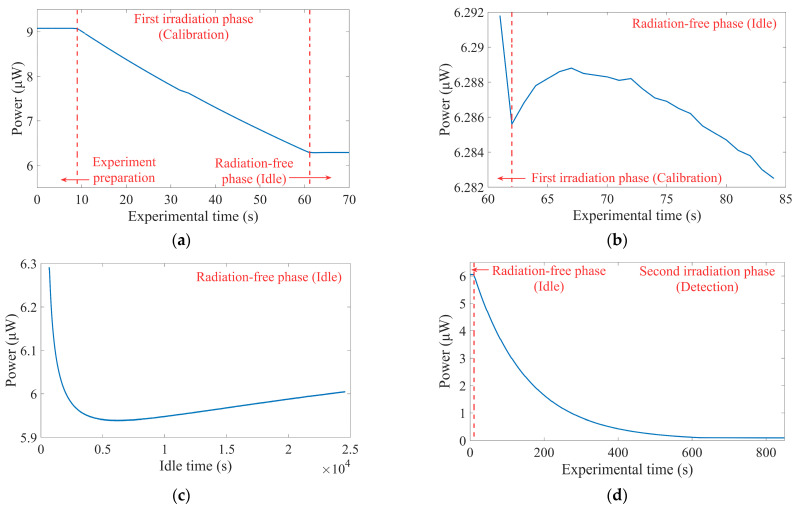
Raw data from the three phases for verification of application method. (**a**) The optical powers in the first irradiation phase with an electron energy of 1 MeV and a dose rate of ~0.498 Gy/s. (**b**) The optical powers at ~20 s at the conclusion of the first irradiation phase. (**c**) The optical powers during the radiation-free phase for ~7 h. (**d**) The optical powers in the second irradiation phase with the same radiation environment as the first irradiation phase.

**Figure 3:**
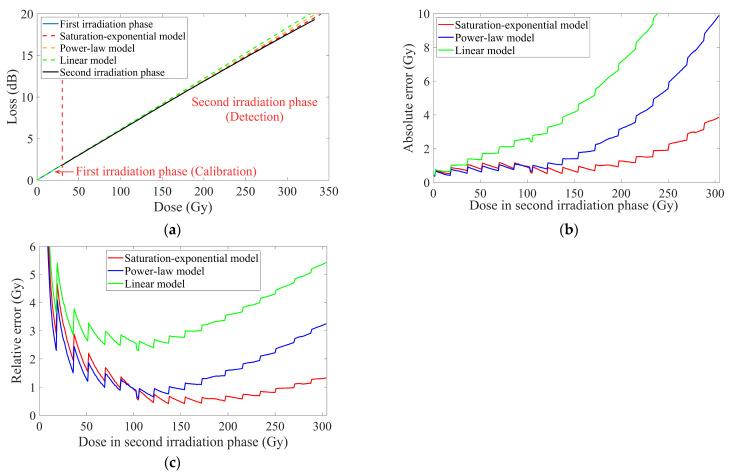
Dose–loss relationships in the two irradiation phases. (**a**) Data from the two phases and the fitting results of the three models applied to the first-phase data. (**b**) Absolute error between the predictions of the three models and the second-phase data. (**c**) Relative errors between the predictions of the three models and the second-phase data.

As illustrated in Figure 2b, transient, rapid, and weak recovery occurred within 3 s after the first irradiation phase, primarily attributed to disruption of the dynamic equilibrium, where the recovery rate of defect centers highly dominated over their negligible generation rate. However, Figure 2c revealed a more pronounced decline over 30 min at the beginning of radiation-free phase. Similar anomalous behavior during recovery phases has been reported in the specific spectral bands of other fibers, though the causal mechanisms remained unexplained [4,61]. Three possible reasons for this phenomenon included: (1) interconversion between defect centers with distinct oscillator strengths, particularly given the coexistence of at least three known defect centers at this wavelength; (2) declining competition between the intensity of photoluminescence and RIA, especially considering a known photoluminescence peak near 625 nm; and (3) temperature-dependent equilibration of defect centers as the temperature of the fiber changed with irradiation or ambient temperature. Identification of the causal mechanisms necessitated high-precision temperature telemetry systems and experimental methodologies such as optically detected magnetic resonance (ODMR). During the final 6 h of the radiation-free phase, the optical power exhibited a gradual upward trend but failed to recover to the value observed at the conclusion of the first irradiation phase. Throughout this period, recovery of defect centers predominated, though the process proceeded at an extremely slow rate under ambient temperature. The initial optical power *P*(0) = 9.0725 μW in Figure 2a was utilized in both the first and second irradiation phases to calculate the loss *L* of transmission light, with the results temporally correlated to radiation doses obtained from the current integrators, as shown in Figure 3a. Models of saturation-exponential, power law, and linearity were employed to fit the dose–loss relationship in the first irradiation phase, and the results are listed in Table 2.

The three models demonstrated near-equal fitting goodness during calibration, failing to select a superior one. Therefore, it was necessary to verify the differences in detection among them. Real-time predicted doses in the second irradiation phase were predicted by applying the second-phase data of losses to the aforementioned fitting models. Therefore, the absolute error *AE* and relative error *RE* between the predicted doses *D_d_* and the experimental data *D_r_* from the current integrators could be calculated using Equation (10):

(10) $$AE=D_{d}-D_{r}\;↔\;RE=\frac{AE}{D_{r}}\times 100\%=\frac{D_{d}-D_{r}}{D_{r}}\times 100\%$$

Through Equation (10), the real-time errors of the fitting models could be derived. These errors could also be plotted with the corresponding detection doses in the second irradiation phase, as further visualized in Figure 3.

The serrated curves observed in Figure 3b,c, characterized by oscillatory fluctuations within small dose intervals, might be attributed to the operational principle of the high-precision electron accelerator. Specifically, the parallel electron beam was generated through repetitive two-dimensional scanning, with the initial position undergoing slight periodic shifts. This process thereby induced very slight and periodic oscillations of dose rate around the preset value. While the Faraday cup with its limited sensing area confined to the central irradiation zone failed to capture such fluctuations, the extended distribution of the sensing fiber along the irradiation zone enabled effective detection of these variations. This observation further suggested the potential utility of such dosimeters in accelerator diagnostics.

All models exhibited zero-bias absolute errors at the onset of detection, causing the relative errors to approach infinity initially. During terminal segment detection, the predictive deviations of three models accelerated due to error accumulation. The power law and linear models exhibited accelerated error escalation starting from the mid-phase of detection. The performance of the saturation-exponential model was similar to that of the power law model in the early phase of detection, but its errors showed the slowest divergence trend in the terminal phase of detection, indicating that it had more sensitively captured and predicted the saturation trend. Overall, the detection accuracy throughout the entire process could be ordered as saturation-exponential > power law > linearity. The absolute errors of the saturation-exponential model were maintained within 1.6 Gy across the detected dose range of 0 to 230 Gy, and the maximum deviations did not exceed 4 Gy, even at terminal segment detection. Furthermore, its relative errors remained below 2.5% within the detected dose range of 40 to 305 Gy. These nice findings validated the feasibility of the application method proposed, particularly given its implementation with commercially available optical fibers.

### 4.2. Sensitivity and Resolution

As derived from Table 2, the linear model yielded a slope *a* = 0.06284 dB·Gy^−1^. Applying Equation (1) and (9) with *l*_0_ = 0.5 m, the RIA-based sensitivity S*_RIA_* of the dosimeter was calculated as 125.6 dB·Gy^−1^·km^−1^. This S*_RIA_* exceeded those reported by some novel sensitive fibers [4,35]. However, considering the disparities in radiation source types, energies, dose rates, and wavelengths during testing of different sensitive fibers, there seemed to be no strong comparability between the specific values reported. This potential enhancement might be attributable to the adoption of a Ge-doped multimode fiber with more active defect center and measurement wavelength. Moreover, the dose rate implemented in our experiments was significantly elevated compared to others.

The dose–loss relationship in Figure 3a exhibited no significant variation in slope. Based on the linear relationship between this slope and S*_RIA_*, the S*_RIA_* of the dosimeter also remained nearly stable throughout the experiment. And in studies of irradiated characteristics, fibers were typically exposed to doses sufficient to induce substantial attenuation.

Selecting the saturation-exponential model for its optimal detection performance, the power-based sensitivity *S_P_* was derived by Equations (3), (5), and (9), as expressed in Equation (11):

(11) $$S_{P}=\varepsilon \frac{\varepsilon P_{0}ln(10)}{10d}{\cdot e}^{\frac{h-D}{d}}\cdot 10^{\frac{\varepsilon }{10}(e^{\frac{h-D}{d}}-1)}$$

**Figure 4:**
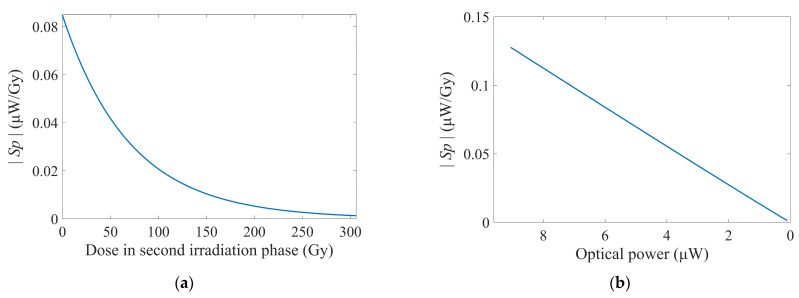
The absolute values of the dosimeter’s power-based sensitivity. (**a**) Correlated with doses in the second irradiation phase. (**b**) Correlated with optical powers in two phases.

All detailed values of parameters were documented in the preceding section. The *S_P_* was calculated as visualized in Figure 4.

The calculated *S_P_* exhibited negative values due to the decline in optical power caused by RIA with increasing doses. We adopted the absolute value |*S_P_*| for analysis. Unlike S*_RIA_*, the |*S_P_*| demonstrated monotonical degradation, rapidly decreasing and asymptotically approaching zero as the doses increased. The |*S_P_*| at the initiation of detection was 0.0847 μW·Gy^−1^. When the cumulative doses reached 50 Gy, |*S_P_*| had declined to approximately half of its initial value. Further progression to 150 Gy resulted in reduction to roughly one-sixth of that at the beginning, and by the finale of the second irradiation phase (~300 Gy), it was diminished to merely 1.6% of its original level, while the loss had not increased to 20 dB. This observation underscored the impracticality of extreme loss levels in the testing of novel fibers, as the detection capability of the dosimeter must be guaranteed throughout its operational range. The dosimeter should be operationally defined as having reached its detection range when |*S_P_*| descended below a predetermined threshold.

During the experiment, |*S_P_*| was also observed to decrease approximately linearly in response to the declining optical power output from the dosimeter. This behavior correlated with the evolution of the optical power shown in Figure 2d, where logarithmic compression in the calculation of *L* caused a large part of the accumulated doses to be mapped into diminished optical power variations in the terminal segment of the curve.

|*S_P_*| was strongly correlated with the precision of the dosimeter. The resolution of Δ*D* could also be derived by Equations (3), (6), and (9), as shown in Equation (12):

(12) $$∆D=∆P\cdot \frac{10d}{Pln\left(10\right)\cdot [\varepsilon +10lg(\frac{P}{P_{0}})]}$$

With the exception of Δ*P*, all detailed values of parameters were also documented in the preceding section. The effect of fluctuation of dark noise on the optical powers during the experiment was observed at approximately 1 nW, exceeding the quantization errors (0.1 nW) from the optical power meter and slim photodiode sensor. Due to the predominance of dark noise in determining the minimum resolvable interval, we consequently defined the Δ*P* as 1 nW. When the radiation-induced decline in optical powers reached 1 nW, the corresponding signal-to-noise ratio SNR or SNR_dB_ could be computed as shown in Equation (13):

(13) $$SNR=\frac{P_{signal}}{P_{noise}}=\frac{∆P}{∆P}=1\;↔\;{SNR}_{dB}=10\mathrm{lg}\left(\frac{P_{signal}}{P_{noise}}\right)=10\mathrm{lg}\left(\frac{∆P}{∆P}\right)=0\;dB$$

**Figure 5:**
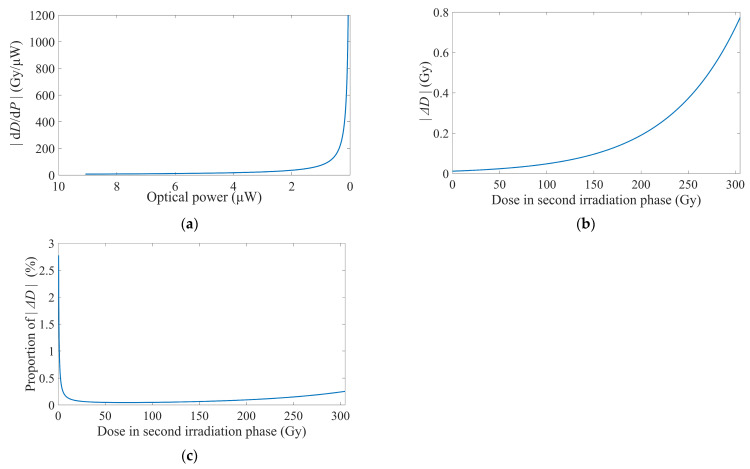
Absolute values of the dosimeter’s resolution. (**a**) Variation of the derivative part. (**b**) Absolute values of resolution correlated with doses in the second irradiation phase. (**c**) Resolution proportion calculated by the absolute values of resolution and doses in the second irradiation phase.

Under this premise, the calculated Δ*D* was illustrated in Figure 5.

Analogous to the behavior of *S_P_*, Δ*D* also exhibited negative values, and we still use their absolute values for analysis. |Δ*D*| demonstrated an accelerated increase with escalating doses. Analysis of the derivative component of |Δ*D*| revealed that continued irradiation, which further reduced optical power, would drive |Δ*D*| to increase even more rapidly, as depicted in Figure 5a. This observation also underscored the necessity of evaluating the operational reliability of dosimeters when sensing fibers are irradiated to excessive loss.

In Figure 5b, |Δ*D*| at the initiation of detection was 0.0118 Gy, with its absolute value remaining below 0.1 Gy throughout the initial cumulative doses of ~150 Gy. Throughout the entire dose range of about ~300 Gy, |Δ*D*| did not exceed 0.8 Gy. Given the reciprocal relationship between the derivative component of |Δ*D*| and Equation (5), the relative amplification of |Δ*D*| also was the reciprocal value of the degradation proportion of |*S_P_*| at an identical dose *D*. Therefore, a detailed listing of |Δ*D*|’s growth with *D* was not provided here. Benefiting from the aforementioned measurement range alongside its resolution, our RIA-based dosimeter exhibited potential applicability as a monitor for food irradiation [62], such as sprout inhibition of potatoes (30–150 Gy), ripening control in fruits (200–500 Gy), and T. spiralis inactivation in pork (≥250 Gy).

In Figure 5c, the proportion of |Δ*D*| represented the calculated |Δ*D*| normalized to the accumulated dose *D* at the same time during the second irradiation phase, thereby directly indicating the detection precision at that dose. The growth rate of |Δ*D*| exceeded that of this proportion, indicating that |Δ*D*| accumulated slower than the dose rate. Throughout detection, the proportion of |Δ*D*| remained below 0.3%, except for at the initial value of 5 Gy. Consequently, the dosimeter maintained an acceptable proportional resolution even at relatively higher levels of radiation.

The analysis in this section was governed solely by the intrinsic sensing properties of RIA-based dosimeters, as manifested through degradation of |*S_P_*| and growth of |Δ*D*|, independent of fiber selection or operational methods. Therefore, rational thresholds for both metrics must be defined to ensure that the dosimeter operated across a dose range with high detection reliability. The proposal of power-based sensitivity and analysis of resolution could provide critical guidance for optimizing design, configuration selection, and operational state assessment of RIA-based dosimeters.

### 4.3. Detectable Range

**Figure 6:**
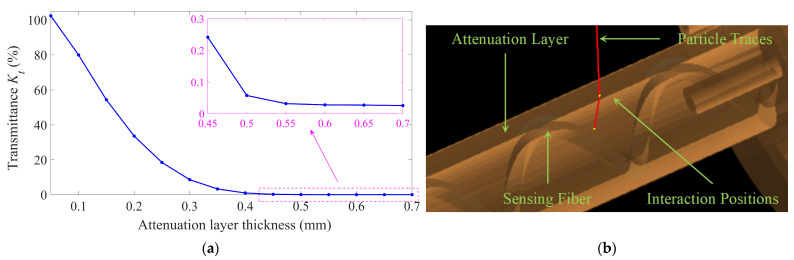
Geant4 simulations of attenuation layers with varying thicknesses under a 1 MeV electron beam. (**a**) Variation in transmittance *K_t_*. (**b**) A particle redirected toward the sensing fiber after interaction.

Prior to experimental validation, Geant4 [63,64,65] simulations were conducted to evaluate the transmission *K_t_* of attenuation layers with varying thicknesses. The sensing probe and fiber were imported into Geant4 via CADMesh (v 1.1). A control group consisting of an identical optical fiber with same material and geometry was positioned under the same parallel beam of radiation, without any shielding layer. The radiation source was configured as an electron beam with the energy of 1 MeV. The thicknesses of the attenuation layers were increased from 0.05 mm to 0.7 mm in 0.05 mm steps, with 10^9^ particles per thickness to ensure statistically robust results. The material of the fibers was configured as silica, while the material of the attenuation layers was defined as copper. Energy depositions from both the sensing and control fibers were processed through Equation (7) to derive *K_t_*, as summarized in Figure 6a.

As anticipated, *K_t_* monotonically decreased with increasing thickness. Notably, *K_t_* exceeded 100% at 0.05 mm, which might be attributed to the statistical nature of Geant4 simulations. As shown in Figure 6b, high-energy particles that should have penetrated geometrical gaps and missed the fibers interacted with the attenuation layer, enabling some primary or secondary particles to be redirected toward the sensing fibers to deposit energy on them. At minimal thicknesses, trajectory redirection dominated over attenuation, causing the numerator in Equation (7) to exceed the denominator. Beyond 0.55 mm, *K_t_* exhibited minimal variation due to the energy deposition from secondary particles (primarily X-rays with stronger penetrating capability) overpowering that contributed by primary electrons.

To adapt the dosimeter for high-dose environments, *K_t_* could be estimated via Equation (8), and thickness selection of copper attenuation layers could be guided as shown in Figure 6a. These simulations validated the feasibility of the range adjustment method proposed in Section 2.4 for RIA-based dosimeters. Of course, the current simulation used the same radiation parameters as the following testing experiments. When the radiation environment changed, *K_t_* simulations under specific application scenarios were required. For example, as the particle energy increased, the thickness of the attenuation layers must be adjusted to ensure that the simulated *K_t_* values remain within the required range. Additionally, when the radiation source changed, the selections of attenuation layers must account for the penetration capability of the particles and avoid materials with peculiar interaction effects.

**Figure 7:**
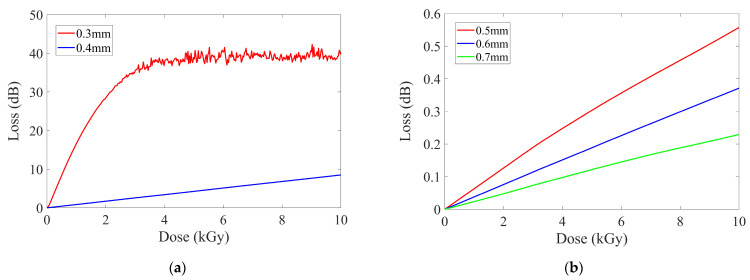
Dose–loss curves with different thicknesses of attenuation layers under the same radiation environment with an electron energy of 1 MeV and a dose rate of ~30 Gy/s until reaching the accumulated dose of ~10 kGy: (**a**) 0.3 and 0.4 mm, (**b**) 0.5–0.7 mm.

After the simulations, copper attenuation layers with thicknesses ranging from 0.3 to 0.7 mm in 0.1 mm increments were tested in field experiments. An identical electron radiation environment with an energy of 1 MeV and a dose rate of ~30 Gy/s until reaching the accumulated dose of ~10 kGy were maintained for the different attenuation layers as long as possible. The dose–loss curves obtained from the experiments were shown in Figure 7.

At 0.3 mm, the terminal segment of the dose–loss curve exhibited erratic fluctuations. Significantly, the power approached almost zero, which meant that the sensing fiber in the probe might have been damaged. For 0.4–0.7 mm, the dose–loss curves exhibited no pronounced entry into nonlinear transition. However, as observed in Figure 7b, all curves demonstrated reduced growth rates in their terminal segments—a premature saturation anomaly inconsistent with theoretical predictions. The accelerator diagnostics revealed an unintentional 6% decline in the dose rate during these experiments, attributed to pressure fluctuations in the ionization chamber, and significantly exceeding the deviation of 0.8% recorded in prior experiments. This variation in the curves proved that the dosimeter captured the changes in dose rate, highlighting its potential for real-time accelerator monitoring. Although the doses from current integrators concurrently decreased, the more pronounced slowdown in losses arose from the dose rate dependency inherent in the sensing fibers.

For the assumption of range adjustment, the detectable upper limits of loss *L*_max_ for identical sensing fibers were approximate. Based on observations of the near-linear region of the dose–loss curve at 0.3 mm, we conservatively assigned this threshold as 15 dB for the current sensing fibers. Therefore, the detectable range could be directly derived from experimental data for 0.3 mm. And for other thicknesses, this prediction of detectable ranges could be calculated by substituting *L*_max_ into the linear models fitted to the experimental data. The decision to avoid saturation-exponential models—despite their superior fitting goodness and predictive capability—stemmed from the aforementioned dose rate decline during experiments. This decline would cause the saturation of such a model to occur prematurely at lower doses, thereby introducing significant errors in the estimation of detectable ranges. The curves in Figure 7 were subjected to curve-fitting and analytical processing, with the results summarized in Table 3.

As anticipated, the predicted detection ranges demonstrated monotonic expansion with increasing thickness of the attenuation layers. This trend validated that RIA-based dosimeters could achieve diverse detectable ranges through tailored attenuation layers without substitution of sensing fiber types.

The relationship between S*_RIA_* and the predicted detection range *R_P_* at any thickness of *i* or *j*, as shown in Table 3, could be approximately expressed by Equation (14):

(14) $$\frac{R_{P,i}}{R_{P,j}}\approx \frac{S_{RIA,j}}{S_{RIA,i}}$$

Equation (14) mathematically underscored that reduced sensitivity constituted an inevitable trade-off for extending the detection range through the selection of attenuation layers.

### 4.4. Repeatability of Different Fibers of the Same Type

**Figure 8:**
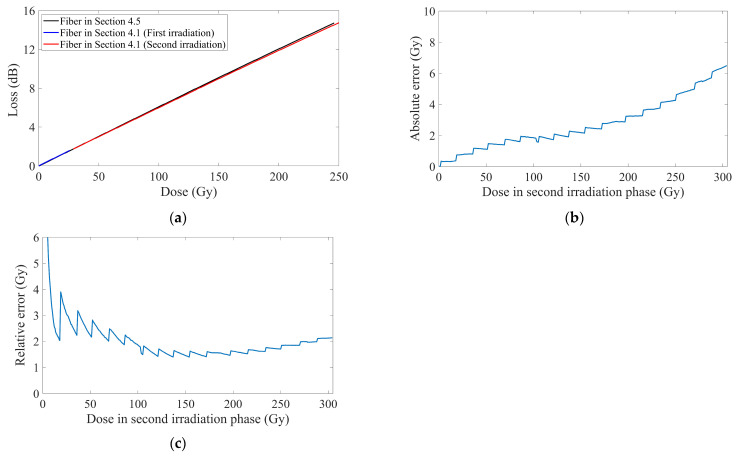
Repeatability verification and application between fibers of the same type and batch. (**a**) Dose–loss curves for two sensing fibers under the same radiation conditions as described in Section 4.1. (**b**) Absolute errors between the predictions of the saturation-exponential model applied to the full-range data in this section and the second-phase data in Section 4.1. (**c**) Relative errors calculated from the absolute errors and second-phase data.

A sensing fiber of the identical type from the same production batch was subjected to continuous irradiation up to ~250 Gy under the same radiation conditions as described in Section 4.1. There is also no attenuation layer, and the dose–loss curve was shown in Figure 8a.

Under identical irradiation conditions, the dose–loss curves of two sensing fibers exhibited close congruence, with a deviation below 1.5%. This consistency substantiated that the same manufacturing process and storage conditions yield minimal batch-to-batch variations in dopant concentrations and native defect densities. Therefore, the full-range data of doses and losses obtained in this section were applied to fit the saturation-exponential model. Utilizing the new results to predict the doses during the second irradiation phase described in Section 4.1, the detection accuracy was demonstrated in Figure 8b,c.

Despite employing fibers of identical type and batch, minor discrepancies still persisted in their responses to RIA. Consequently, utilizing the full-range fitting results from one fiber to predict the detection doses of the other inevitably introduced additional errors. As evidenced by comparison of Figure 3 and Figure 8, the detection accuracy achieved in this section was markedly inferior to that of the saturation-exponential model via detection after calibration in Section 4.1, but close to that of the power law model. Moreover, the current accuracy in this section prominently surpassed the performance of the linear models in Section 4.1. This phenomenon indicated sufficiently high RIA response consistency within the same batch, where the errors induced by fiber-to-fiber variations became less significant than those arising from the inherent limitations of the models’ fitting capabilities.

As analyzed in Section 4.2, calibration might consume the starting segment of optical power, which possesses better sensitivity and resolution. To maximize utilization of this segment, implementing a full-range fitting result from fibers of identical type and batch presented a viable method to bypass calibration. However, using such a method, the minor differences between the two fibers could not be described with universal patterns, and the newly introduced errors might be impossible to be estimated or fully compensated. Prior research [4,66] further cautioned that, when employing sensing fibers of the same type but from different production batches, their models still required individual calibration and analysis.

### 4.5. Discussion of Other Factors

During the experiments, we maximized efforts to prevent significant interference from temperature on the core conclusions of this work. The environmental temperature was stringently maintained at 15–17 °C. Furthermore, we have planned to design a direct connection between the inner core and a thermostat. This might proactively address two objectives: (1) maintaining precise temperature stability during experiments to eliminate interference variables, and (2) setting a foundation for subsequent investigations into temperature-driven effects on RIA-based dosimeters.

To minimize the size and weight of the sensing probes, a fiber has been adopted with a reduced coiling radius, resulting in ~30% macro-bend loss [67] on transmission of light. Undesirable loss of optical power degraded SNR, also reducing the resolution of the system. Concurrently, the reduced coiling radius might induce additional stress in the sensing fiber, causing minor variations in defect concentration [3]. Furthermore, studies have suggested that mode field displacement from a tight coiling radius—when coupled with differential defect concentrations in the core and cladding of the fiber under radiation—might generate more complex response variations of RIA in Ge-doped fibers [68]. Therefore, quantitative analysis of these variations under differing macro-bends necessitated further mechanism investigations and experimental tests.

**Figure 9:**
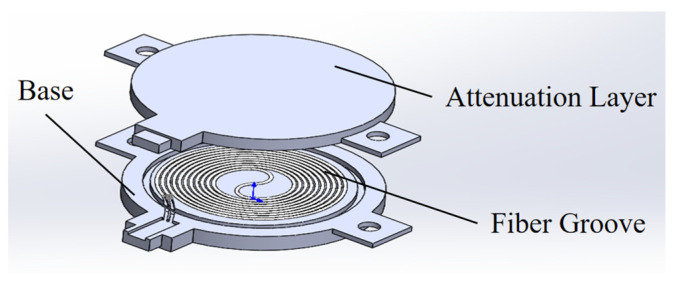
Sensing probe with a planar structure.

For further optimization in application, the solid core might be replaced by a hollow core with an increased radius. Alternatively, a planar structure as illustrated in Figure 9 might serve as the sensing probe, with an expanded radius for the inner fiber. However, the base should be of a certain thickness to meet strength requirements, inevitably attenuating or even overshadowing incidental radiation. Consequently, planar sensing probes are typically limited to hemispherical radiation detection.

The apparatus constructed in this study primarily aimed to validate the proposed application method for an RIA-based dosimeter with common fibers and an analysis method for its sensing performance. The selection of widely available sensing fibers and the low-cost miniaturized design collectively facilitated the commercialization and deployment of such dosimeters in specific scenarios. Obviously, adopting more sophisticated and expensive components could further optimize its performance or expand its functionalities. For example, utilizing novel fibers with reduced temperature/dose rate dependency could ensure that it can be employed in highly dynamic radiation environments [21,34], and implementing OTDR could achieve distributed sensing via single fiber [4,37]. However, such enhancements inevitably escalated the costs, dimensions, or weight, while potentially compromising operational reliability and longevity.

Considering that RIA could be generated by various radiation types, such as gamma [4], X-ray [35], and neutron [69], the dosimeter developed in this work could also be applicable to other radiation types. The metrics proposed could analyze the sensing performance of RIA-based dosimeters under such radiation types.

## 5. Conclusions

In this work, we proposed an application method for RIA-based dosimeters constructed from commercial fibers and analytical methods for all such dosimeters. Then, all the methods were validated through a low-cost and miniaturized validation apparatus using commercial fibers.

To overcome the prevalent inherent dependencies of defect centers, we proposed a calibration-detection workflow under an identical radiation environment. Through this application method, the RIA-based dosimeters could achieve repetitive measurements operating in thermostatic and radiation-stable environments even by simple replacement of commercial fibers. Comparative analysis of three fitting models revealed the following detection accuracy under our design: saturation-exponential > power law > linearity. Applying the saturation-exponential model, the dosimeter demonstrated absolute errors below 4 Gy within ~300 Gy after approximately 30 Gy calibration. These promising results demonstrated that commercial optical fibers could serve as viable sensing candidates in specific application scenarios. This progress would broaden fiber selection while drastically reducing the cost of RIA-based dosimeters, which might enable commercialization and further application. Specifically, RIA-based dosimeters with commercial fibers might provide an inexpensive solution with low operational difficulty for repetitive monitoring in well-defined radiation environments, such as dose detection for food irradiation and radiotherapy. Notably, the dosimeter successfully captured subtle dose rate fluctuations during the experiments, demonstrating its potential for stability monitoring in accelerator facilities.

Subsequently, we proposed quantification procedures for power-based sensitivity and resolution. Distinct from conventional RIA-based sensitivity, which is primarily focused on material properties, these metrics directly characterized progressive degradation of the response ratio and precision during detection. Importantly, this analytical method remained universally applicable to all RIA-based dosimeters, regardless of component or fiber selection, providing critical guidance for design optimization and operational assessment. Verification of RIA response consistency across fibers of the same type and batch further supported the feasibility of sharing parameters in the fitting model, potentially eliminating per-calibration requirements for every fiber.

For high-dose applications, we developed an optimization method by setting up attenuation layers to adjust the detectable ranges of RIA-based dosimeters. Both Geant4 simulations and experimental data under 1 MeV electron irradiation confirmed that incrementally increasing the thickness of the copper attenuation layers enabled monotonic expansion of detectable doses. This thickness- or material-tunable design allowed flexible range selection without the replacement of fiber types, significantly broadening application scenarios with low operational difficulty. In the final section, we also briefly discussed the temperature control, structural designs, and scalability of the validation apparatus.

While current research on RIA-based dosimeters has predominantly focused on the development and full testing of novel fibers, this work provided insights into the engineering-oriented popularization, optimization, and functional expansion of such dosimeters through a new application method and performance characterization. Particularly given the availability, affordability, and user-friendliness of commercial optical fibers, RIA-based dosimeters constructed with them might emerge as an intriguing candidate in various radiation-related areas.

## Figures and Tables

**Table 1:** Characteristics of the OM2 GRIN fibers, given by Corning.

Fiber Parameter	Value
Numerical Aperture	0.22
Core Diameter	50 µm
Cladding Diameter	125 µm
Coating Diameter	0.9 mm
Losses @800 nm	<3.5 dB/km

**Table 2:** Fitting results of the three models applied to the first-phase data.

Models	Results	R-Squares
Saturation-exponential	$L=236.5\times (1-e^{\frac{-0.1630-x}{2864}})$	0.9998
Power law	$L=0.0628\times D^{0.9914}+0.0064$	0.9998
Linearity	L=0.0610×D+0.0108	0.9998

**Table 3:** Fitting results and data analysis for different thicknesses.

Thicknesses (mm)	Fitting Results	R-Squares	S*_RIA_* (dB·km^−1^·Gy^−1^)	Detectable Ranges * (kGy)
0.3	L=17.43×D−0.4340	0.9993	34.86	0.8943
0.4	L=0.8513×D+0.0061	1.000	1.703	17.61
0.5	L=0.0552×D+0.0174	0.9974	0.1104	271.4
0.6	L=0.0371×D+0.0018	0.9999	0.0743	403.8
0.7	L=0.0224×D+0.0035	0.9998	0.0449	647.2

* The detectable ranges were predicted by linear models with *L*_max_ = 15 dB.

## Data Availability

The raw data supporting the conclusions of this article will be made available by the authors on request.
